# Visceral Adiposity Index (VAI) Is Predictive of an Altered Adipokine Profile in Patients with Type 2 Diabetes

**DOI:** 10.1371/journal.pone.0091969

**Published:** 2014-03-20

**Authors:** Marco C. Amato, Giuseppe Pizzolanti, Vittoria Torregrossa, Gabriella Misiano, Salvatore Milano, Carla Giordano

**Affiliations:** 1 Dipartimento Biomedico di Medicina Interna e Specialistica (Di.Bi.M.I.S.), Section of Endocrinology, Diabetology and Metabolism, University of Palermo, Palermo, Italy; 2 Dipartimento di Biopatologia e Biotecnologie Mediche e Forensi (Di.Bi.Me.F), Section of Clinical Pathology, University of Palermo, Palermo, Italy; Hosptial Infantil Universitario Niño Jesús, CIBEROBN, Spain

## Abstract

**Aims:**

Although there is still no clear definition of “adipose tissue dysfunction” or ATD, the identification of a clinical marker of altered fat distribution and function may provide the needed tools for early identification of a condition of cardiometabolic risk. Our aim was to evaluate the correlations among various anthropometric indices [BMI, Waist Circumference (WC), Hip Circumference (HC), Waist/Hip ratio (WHR), Body Adiposity Index (BAI) and Visceral adiposity Index (VAI)] and several adipocytokines [Visfatin, Resistin, Leptin, Soluble leptin receptors (sOB-R), Adiponectin, Ghrelin, Adipsin, PAI-1, vascular endothelial growth factor (VEGF), Hepatocyte growth factor (HGF) TNF-α, hs-CRP, IL-6, IL-18] in patients with type 2 diabetes (DM2).

**Materials and Methods:**

Ninety-one DM2 patients (age: 65.25±6.38 years; 42 men and 49 women) in stable treatment for the last six months with metformin in monotherapy (1.5–2 g/day) were cross-sectionally studied. Clinical, anthropometric, and metabolic parameters were evaluated. Serum adipocytokine levels were assayed with Luminex based kits.

**Results:**

At the Pearson’s correlation, among all the indices investigated, VAI showed a significant correlation with almost all adipocytokines analyzed [Visfatin, Resistin and hsCRP (all p<0.001); Adiponectin, sOb-R, IL-6, IL-18, HGF (all p<0.010); Ghrelin and VEGF (both p<0.05)]. Through a two-step cluster analysis, 55 patients were identified with the most altered adipocytokine profile (patients with ATD). At a ROC analysis, VAI showed the highest C-statistic [0.767 (95% CI 0.66–0.84)] of all the indices.

**Conclusions:**

Our study suggests that the VAI, among the most common indexes of adiposity assessment, shows the best correlation with the best known adipocytokines and cardiometabolic risk serum markers. Although to date we are still far from clearly identifying an ATD, the VAI would be an easy tool for clearly mirroring a condition of cardiometabolic risk, in the absence of an overt metabolic syndrome.

## Introduction

Metabolically complicated obesity and its associated insulin resistance are increasingly prevalent and contribute to increasing the prevalence of type 2 diabetes and cardiovascular disease [1]–[3], two major causes of morbidity and mortality in our population. Excessive triglyceride storage in adipocytes, due to weight increase, is known to induce changes in adipose tissue (AT), such as adipocyte insulin resistance (resulting in higher lipolytic activity), decreased adiponectin production, increased TNF-α, IL-6 production and increase in several other adipocytokines. These changes are characteristic of “adipose tissue dysfunction” (ATD) or “adiposopathy” [4] which is often associated with diabetes mellitus, hypertension and diabetic dyslipidemia [low high-density lipoprotein cholesterol (HDL-C) and raised triglycerides]. However, this condition is not necessarily associated with obesity; it can occur with a mild increase in body weight, even within the nonobese range [5], [6]; conversely, the threshold may not be reached even in the presence of obesity [7]. Therefore, the existence of simple tools for clinical evaluation of this condition would allow better identification of people at risk for systemic metabolic complications and lay the foundation for developing more effective strategies to prevent such complications as ectopic fat deposition and systemic insulin resistance.

In 2010 we modelled the Visceral adiposity index (VAI), a gender-specific mathematical index based on simple anthropometric [BMI and waist circumference (WC)] and metabolic [Triglycerides (TG) and HDL Cholesterol (HDL)] parameters, as a presumed surrogate marker of adipose tissue function and distribution, independently linked to insulin sensitivity and cardiometabolic risk in the general population [8]. In the last three years, the VAI has been reported on in 30 publications, in which its capacity to express cardiometabolic risk and possible ATD was identified.

The most important results were obtained in populations at metabolic risk not always having an overt metabolic syndrome, such as the general population [9]–[12], post-menopausal women [13] and women with Polycystic Ovary Syndrome [14]. Recently, in a population of young women with PCOS, the VAI proved to be able to replace visceral Computed Tomography scanning as a marker for visceral adiposity and to predict insulin resistance [15]. These findings have led to the proposal of a precise VAI cut-off to differentiate the “metabolically healthy polycystic ovary syndrome” from the “metabolically unhealthy polycystic ovary syndrome” [16].

Also, in patients with non-alcoholic fatty liver disease, the VAI is linked to significant fibrosis [17], [18], while it is not more powerful than WC in discriminating steatosis from steatohepatitis [19]; in patients with HCV, the VAI showed an independent association with both steatosis and necroinflammatory activity and has a direct correlation with viral load [20]; in patients with acromegaly, the VAI appears to be associated with disease activity, adiponectin levels and insulin sensitivity and secretion, and is influenced independently of GH levels [21].

However, in all these studies the ability of the VAI to express the adipose tissue function was only a hypothesis, few studies existing that have evaluated the correlation between it and levels of adipocytokine [9], [17], [21].

The aim of this study is to evaluate the relationship between the VAI and other common anthropometric measures of fat distribution [BMI, WC, Hip Circumference (HC), Waist-hip ratio (WHR), Body Adiposity Index (BAI)], on the one hand, and a complete panel of adipocytokines [Resistin, Leptin, Soluble Leptin Receptor (sOB-R), Leptin/sOB-R ratio, Adiponectin, Visfatin, Ghrelin, Adipsin, PAI, Vascular-endothelial growth factor (VEGF), TNF-α, hs-CRP, IL-6, IL-18, Hepatocyte growth factor (HGF)], on the other, in patients with type 2 diabetes mellitus (DM2).

## Materials and Methods

### Subjects

This study was approved by the Institutional Review Board at the Faculty of Medicine of the University of Palermo. At the time of observation all patients regularly signed an informed consent for the scientific use of their data.

Ninety-one consecutive Caucasian patients with DM2 (mean age 62.25±6.38 years; range: 51–75), followed up in our dedicated Outpatients Clinic (from January 1st 2011 to December 31st 2012), were cross-sectionally studied. Inclusion criteria were a 2-year or longer history of DM2 at study entry, in stable treatment for the last six months with metformin (1.5–2 g/day). Only patients were included who had HbA1c ≤8 (64 mmol/mol), in order to avoid the effects of severe glucotoxicity on the adipocytokines. The following were excluded: patients with type 1 diabetes, patients with previous treatment with other anti-diabetic drugs in the past six months, patients with morbid obesity, pendulous abdomen, severe hypertriglyceridemia and/or or use of fibrates [22]. Also excluded were patients with macro- or microvascular complications (except 7 patients with simple preproliferative retinopathy). Patients were not excluded that had essential hypertension (58 patients). In the patients included a complete medical history, a complete physical examination, body weight, height, waist and hip circumference were recorded. Waist circumference (WC) was measured midway between the inferior margin of the last rib and the crest of the ilium in a horizontal plane by the measurer sitting by the subject and fitting the tape snugly, but not compressing soft tissues. Hip circumference (HP) was measured around the pelvis at the point of maximal protrusion of the buttocks. Fasting blood samples and a morning spot urine sample were collected for biochemical analyses. All participants were instructed to perform home glucose monitoring (one full day glucose profile per week: fasting, 2 h after breakfast, 2 h after lunch and 2 h after dinner).

### Anthropometric Indices

BMI was calculated as body weight (in kilograms) divided by the square of height (in meters). WHR and was calculated by dividing the waist circumference by the hip circumference. Body adiposity Index (BAI) was calculated using the following formula [23]:
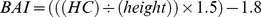



HC is expressed in centimeters and height in meters.

The VAI was calculated as described [8], [22] using the following sex-specific equation, where TG is triglyceride levels expressed in mmol/l and HDL is HDL-cholesterol levels expressed in mmol/l:
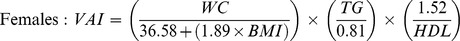





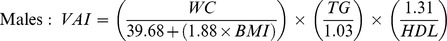



### Assay

The BioPlex Pro Human Diabetes 10-plex assay (BioRad, Milan, Italy) was used to quantitate the following diabetes-related analytes: C-peptide (nmol/l), Ghrelin (pg/ml), Leptin (ng/ml), PAI-1 (ng/ml), Resistin (ng/ml) and visfatin (pg/ml). The BioPlex Pro Human Diabetes Adipsin and Adiponectin duplex assay (BioRad, Milan, Italy) was used for Adipsin (ng/ml) and Adiponectin (μg/ml) assays. A customized BioPlex 3-plex assay (BioRad, Milan, Italy) was used for the quantitation of serum IL-6 (pg/ml), TNF-α (pg/ml) and VEGF pg/ml. A customized BioPlex duplex assay (BioRad, Milan, Italy) was used for the quantitation of IL-18 (pg/ml) and HGF (pg/ml). All these assays were run on a BioPlex 200 workstation (BioRad, Milan, Italy). Human sOb-R (ng/ml) was assayed using an ELISA sandwich enzyme immunoassay (Human leptin receptor ELISA, BioVendor, Heidelberg, Germany). Serum non-esterified fatty acids (NEFA) (mmol/l) were assayed after overnight fasting using an enzymatic colorimetric method (Randox NEFA assay FA115, Randox Laboratories, County Antrim, UK). CRP (mg/l) was determined by a high sensitivity immunonephelometric method (CardioPhase hs CRP, SIEMENS) on the BNTN II System (SIEMENS). Blood glucose levels (mg/dl) were measured using an electrochemical system (Glucocard, Menarini Diagnostics, Italy). Total cholesterol, HDL cholesterol, triglycerides and urinary albumin and creatinine were measured in our laboratory using standard assays. HbA1c was determined by High Pressure Liquid Chromatography (HPLC) with ion-exchange resin (HA8121, Hi-AutoA1c, Menarini, Italy). LDL cholesterol levels were calculated with Friedewald’s formula. The conversion factors for the International System (SI) were the following: glucose (mg/dl vs. mmol/l; X 0.0555), insulin (mUI/l vs. pmol/l; X 6.945), total cholesterol (mg/dl vs. mmol/l; X 0.0259), visfatin (pg/ml vs. ng/ml; :1000), Ghrelin (pg/ml vs pmol/l; X 0.296), Adipsin (ng/ml vs. μg/ml; :1000).

### Statistical Methods

The Statistical Packages for Social Sciences SPSS version 17 and MedCalc version 11.3 were used for data analysis. Baseline characteristics were presented as mean ± Standard Deviation (SD) for continuous variables; rates and proportions were calculated for categorical data. Normality of distribution for quantitative variables was assessed by the Shapiro-Wilk test. Some variables (Leptin, Leptin/sOb-R ratio, Adiponectin, Resistin, TNF-α, IL-18 and hs-CRP) did not show normal distribution, and the natural logarithmic transformed (Ln) values of each adipocytokine for statistical analysis were used. Differences between the two groups were detected by the unpaired Student’s t test for continuous variables (after testing for equality of variance: Levene test) and by the χ2-test and Fisher’s exact test (when appropriate) for categorical variables. Correlations among anthropometric indices and adipocytokines were analysed using the Pearson’s test.

In order to identify patients with Hypothetical ATD (HATD), we performed a two-step cluster analysis using log-likelihood distance measures. This technique can detect latent relationships within a complex data set between patients with multiple distinct characteristics. Cluster analysis was applied using an a priori number of fixed clusters (cluster 1 and cluster 2) and the following continuous variables were included in the analysis: Ln Leptin/sOb-R ratio, Ln Adiponectin, Ln Resistin, Ln NEFA and Ln TNF-α. The selection of the variables included in the analysis was made on the basis of the Silhouette Coefficient obtained by including various combinations of adipocytokines. The 5 variables included produced a Silhouette Coefficient >0.5, indicative of a good partitioning of data. The two clusters identified were designated as “normal adipose tissue function” (cluster 1) and HATD (cluster 2). For comparison between cluster 1 and cluster 2, the group sizes gave 74.84% power to detect a moderate effect size (Cohen’s d = 0.5) using T-test, with alpha at 0.05. Post-hoc power analysis was performed using G*Power Version 3.1.6 software. The receiver-operating characteristic (ROC) curve analysis was conducted using the MedCalc software v. 9.3.8.0 for Windows [which uses calculation of the area under the curve (C-statistic) and 95% confidence intervals by the technique of Hanley and McNeil], to evaluate the diagnostic performance of the various anthropometric indices (BMI, WC, HC, WHR, BAI, VAI) for predicting HATD. The differences between C-statistics were calculated by the method of Hanley and McNeil.

A p value of <0.05 was considered statistically significant.

## Results

The 91 patients studied consisted in 49 women and 42 men; the clinical characteristics, the glycometabolic state and the adipocytokine pattern are summarized in Table 1. Regarding the anthropometric indices, the women showed a higher BMI (30.35±4.94 vs. 28.36±4.09 kg/m^2^, p = 0.039), HC (110.30±11.66 vs. 103.66±7.40 cm, p = 0.001), BAI (37.09±6.27 vs. 30.31±4.17, p<0.001), VAI (2.64±1.56 vs. 1.87±0.94, p = 0.005) and a lower WHR (0.93±0.10 vs. 0.98±0.04, p = 0.012). Regarding the adipocytokine profile, only significantly higher levels of leptin (13.74±12.02 vs. 3.59±4.79 ng/dl, p<0.001) and significantly lower levels of sOb-R (19.89±6.18 vs. 23.10±6.40 ng/ml, p = 0.018) were found in women as compared to men. No other gender difference was found.

**Table 1:** Clinical characteristics and pattern of adipocytokine secretion in the patients with type 2 diabetes.

	Women	Men	Total
	No 49	No 42	No 91
	*Mean ± SD*	*Mean ± SD*	*Mean ± SD*
Age (years)	62.37±6.67	62.12±6.10	62.25±6.38
Duration of disease (years)	9.53±6.50	9.95±5.40	9.73±5.99
BMI (Kg/m^2^)	30.35±4.94*	28.36±4.09	29.43±4.65
Waist Circumference (cm)	103.02±12.43	101.93±10.10	102.52±11.36
Hip Circumference (cm)	110.30±11.66*	103.66±7.40	107.24±10.42
Waist/Hip ratio	0.93±0.10*	0.98±0.04	0.95±0.08
Body Adiposity Index	37.09±6.27**	30.31±4.17	33.96±6.35
Visceral Adiposity Index	2.64±1.56*	1.87±0.94	2.29±1.36
HbA1c (%)	6.94±0.68	6.88±0.57	6.92±0.63
HbA1c (mmol/mol)	52±7.40	52±6.20	52±6.90
HOMA-IR	2.98±3.02	3.26±3.44	3.11±3.21
Fasting Insulin (pmol/l)	61.75±63.33	68.94±72.31	65.07±67.33
Fasting C-peptide (nmol/l)	0.14±0.16	0.16±0.15	0.15±0.15
Urinary Albumin-to-Creatinine Ratio (UACR) (mg/g)	16.13±47.53	17.05±26.31	16.55±38.99
**Fasting lipids**			
Total Cholesterol (mmol/l)	4.67±0.78	4.53±0.97	4.60±0.87
HDL Cholesterol (mmol/l)	1.31±0.31	1.20±0.32	1.25±0.32
LDL Cholesterol (mmol/l)	2.75±0.82	2.79±0.91	2.77±0.86
Triglycerides (mmol/l)	1.45±0.61	1.36±0.51	1.41±0.57
NEFA (mmol/l)	0.54±0.27*	0.39±0.24	0.47±0.26
**Fasting adipocytokines**			
Leptin (ng/dl)^§^	13.74±12.02**	3.59±4.79	9.06±10.65
Soluble leptin receptor (sOb-R) (ng/ml)	19.89±6.18*	23.10±6.40	21.37±6.46
Leptin/sOb-R ratio^§^	0.89±1.09**	0.20±0.34	0.57±0.90
Adiponectin (μg/ml)^ §^	10.95±12.10	13.24±14.37	12.01±13.17
Visfatin (ng/ml)	0.64±0.56	0.61±0.50	0.62±0.53
Resistin (ng/ml)^ §^	3.78±4.36	4.42±5.28	4.07±4.79
Ghrelin (pmol/l)	31.49±9.96	30.83±7.66	31.19±8.93
Adipsin (μg/ml)	0.63±0.35	0.70±0.30	0.67±0.33
PAI-1 (ng/ml)	83.51±78.96	83.78±86.79	83.63±82.20
TNF-α (pg/ml)^ §^	1.43±1.62	1.49±1.64	1.46±1.62
IL-6 (pg/ml)	2.24±2.42	2.21±2.18	2.22±2.30
IL-18 (pg/ml)^ §^	41.75±27.32	39.13±24.47	40.54±25.93
VEGF (pg/ml)	106.26±90.91	117.81±104.69	111.59±97.13
HGF (pg/ml)	351.82±185.87	385.22±209.67	367.23±196.80
hs-CRP (mg/l)^ §^	0.50±0.67	0.97±4.44	0.72±3.04

Women vs. Men:

* p<0.05,

**p<0.001; Student’s t Test after testing for equality of variance (Levene test) and after natural log transformation of several variables (§).

### Comparison of Various Anthropometric Indices with the Adipocytokines, Lipid and Glycemic Parameters

#### BMI

BMI showed a significant positive correlation with Ln Leptin, Ln Leptin/sOb-R ratio, HOMA IR, fasting C-peptide and fasting Insulin; by contrast, a significant inverse correlation was observed with sOb-R and Ghrelin (Table 2).

**Table 2:** Bivariate correlations between anthropometric measures and adipocytokine, lipid and glycemic parameters.

	BMI	WC	HC	WHR	BAI	VAI
**ADIPOCYTOKINES**						
Ln Leptin	0.454***	0.400***	0.535***	−0.045	0.542***	0.239*
sOb-R	−0.436***	−0.412***	−0.300**	−0.204	−0.239*	−0.278**
Ln Leptin/sOb-R ratio	0.491***	0.435***	0.526***	0.008	0.521***	0.270**
Ln Adiponectin	−0.002	0.053	0.109	−0.060	0.006	−0.330**
Visfatin	0.116	0.115	0.093	0.036	0.087	0.375***
Ln Resistin	0.045	0.217*	0.103	0.156	−0.028	0.379***
Ghrelin	−0.228*	−0.296**	−0.108	−0.263*	−0.045	−0.225*
Adipsin	−0.008	0.115	0.078	0.055	0.004	0.025
PAI-1	0.013	0.107	0.008	0.153	−0.004	0.086
Ln TNF-α	−0.007	0.003	−0.206	0.213*	−0.180	0.170
IL-6	0.145	0.187	0.015	0.233*	0.083	0.300**
Ln IL-18	−0.052	−0.095	0.001	−0.134	0.076	0.340**
VEGF	0.072	0.097	−0.097	0.250*	−0.197	0.211*
HGF	0.076	0.200	0.065	0.177	0.039	0.298**
Ln hs-CRP	0.155	0.233*	0.171	0.120	0.158	0.419***
**LIPID PATTERN**						
Total Cholesterol	−0.082	−0.165	−0.078	−0.122	0.046	0.316**
HDL Cholesterol	−0.087	−0.065	0.113	−0.194	0.145	–
Triglycerides	0.107	0.051	0.069	0.003	0.115	–
LDL Cholesterol	−0.075	−0.152	−0.144	−0.041	−0.062	0.286**
NEFA	0.060	−0.011	0.042	−0.051	0.205	0.327**
**GLYCEMIC PATTERN**						
HOMA IR	0.373***	0.465***	0.421***	0.122	0.236*	0.324**
Fasting C-Peptide	0.228*	0.250*	0.325**	−0.028	0.129	0.228*
Fasting Insulin	0.336**	0.449***	0.426***	0.097	0.220*	0.329**
Fasting glucose	0.184	0.262*	0.098	0.216*	0.075	0.031
2 h after breakfast glucose	−0.067	0.026	−0.105	0.162	−0.076	0.166
2 h after lunch glucose	0.024	0.089	0.006	0.089	−0.037	0.069
2 h after dinner glucose	0.070	0.157	0.094	0.078	0.085	0.128
HbA1c	0.127	0.268*	0.132	0.202	0.125	0.250*

BMI: Body Mass Index; WC: Waist Circumference; HC: Hip Circumference; WHR: Waist/Hip ratio; BAI: Body Adiposity Index; VAI: Visceral adiposity Index; sOb-R: Soluble leptin receptor; PAI-1: Plasminogen activator inhibitor-1; TNF-α: Tumor necrosis factor alpha; IL-6: Interleukin 6; IL-18: Interleukin 18; VEGF: Vascular endothelial growth factor; HGF: Hepatocyte growth factor; Hs-CRP: high-sensitivity C reactive protein.

Bivariate Pearson correlation: the value indicates *r coefficient.*

*p<0.050;

**p<0.010;

***p<0.001.

#### WC

WC showed a significant positive correlation with Ln Leptin, Ln Leptin/sOb-R ratio, Ln Resistin, Ln hs-CRP, HOMA IR, fasting C-peptide and fasting Insulin, fasting Glucose and HbA1c; a significant inverse correlation was observed with sOb-R and Ghrelin (Table 2).

#### HC

HC, excluding Ghrelin, showed similar significant correlations to BMI (Table 2).

#### WHR

WHR significantly correlated with Ghrelin, Ln TNF-α, IL-6, VEGF and fasting glucose.

#### BAI

BAI showed significant correlations only with Ln Leptin, sOb-R, Ln Leptin/sOb-R ratio, HOMA IR and fasting Insulin.

#### VAI

VAI is the anthropometric index that showed the largest number of correlations with adipocytokines. Significant positive correlations were found with Ln Leptin, Ln Leptin/sOb-R ratio, Visfatin, Ln Resistin, IL-6, Ln IL-18, VEGF, HGF, Ln hs-CRP, Total Cholesterol, LDL Cholesterol, NEFA, HOMA IR, fasting C-peptide and fasting Insulin. Significant inverse correlations were observed with sOb-R, Ghrelin and Ln Adiponectin.

### Two-Step Cluster Analysis

**Figure 1:**
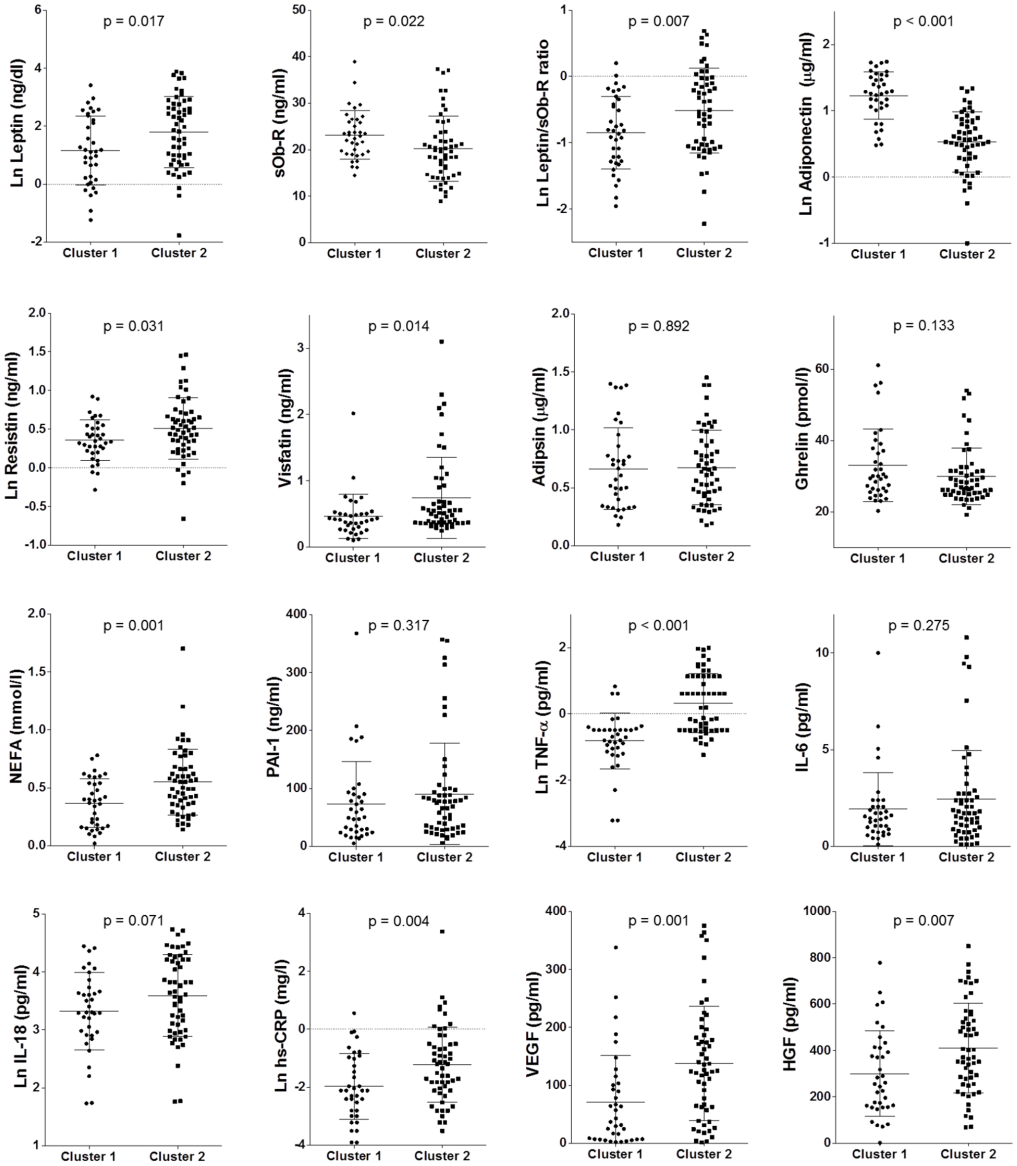
Differences in serum levels of adipocytokines between Cluster 1 (DM2 patients without “adipose tissue dysfunction”) and Cluster 2 (DM2 patients with “hypothetical adipose tissue dysfunction”).

In order to identify a group of patients with the most altered adipocytokine profile, classifiable as patients with HATD, a two-step cluster analysis was performed (based on the selection of Ln Leptin/sOb-R ratio, Ln Adiponectin, Ln Resistin, Ln NEFA and Ln TNF-α. Two clusters were identified (Cluster 1 = 36 patients; Cluster 2 = 55 patients) of which cluster 2 showed the worst adipocytokine profile (Figure 1). Regarding the specific parameters of diabetes (duration of disease, HbA1c, HOMA IR, glycemic profile), there were no significant differences between the two clusters (Table 3). Significant differences were observed for lipids and adipocytokines. Indeed, cluster 2 showed significantly higher levels of triglycerides (1.58±0.61 vs. 1.14±0.38 mmol/l; p<0.001), NEFA (0.54±0.28 vs. 0.36±0.20 mmol/l, p = 0.001) and lower levels of HDL cholesterol (1.14±0.22 vs. 1.43±0.37 mmol/l, p<0.001); no difference was found for total cholesterol and LDL cholesterol. Furthermore, cluster 2 identified patients with higher levels of Ln Leptin (1.78±1.22 vs. 1.15±1.18 ng/dl, p = 0.017), Ln Leptin/sOb-R ratio (− 1.16±1.46 vs.–1.96±1.20, p = 0.007), Ln Resistin (1.17±0.91 vs. 0.82±0.60 ng/ml, p = 0.031), Visfatin (0.74±0.61 vs. 0.45±0.33 ng/ml, p = 0.014), Ln TNF-α (0.32±0.89 vs.–0.81±0.84 pg/ml, p<0.001), Ln hs-CRP (− 1.22±1.29 vs.–1.97±1.32, p = 0.004), VEGF (138.13±98.53 vs. 71.04±80.51 pg/ml, p = 0.001), HGF (411.45±193.95 vs. 299.67±183.82 pg/ml, p = 0.007) and lower levels of sOb-R (20.20±6.98 vs. 23.16±5.16 ng/ml, p = 0.022) and Ln Adiponectin (1.17±1.22 vs. 2.83±0.81 μg/ml, p<0.001). No significant differences were found for Adipsin, Ghrelin, PAI-1, IL-6 and IL-18.

**Table 3:** Differences in anthropometric and glycometabolic characteristics between cluster 1 and cluster 2.

	Cluster 1	Cluster 2	
	No 36	No 55	
	*Mean ± SD*	*Mean ± SD*	*p*
Age (years)	62.83±5.48	61.87±6.92	0.486
Duration of disease (years)	10.56±6.45	9.18±5.66	0.301
BMI (Kg/m^2^)	28.33±4.08	30.15±4.90	0.060
Waist Circumference (cm)	100.36±12.07	103.93±10.76	0.155
Hip Circumference (cm)	107.61±11.19	107±9.98	0.791
Waist/Hip ratio	0.93±0.08	0.97±0.08	0.036
Body Adiposity Index	33.27±6.53	34.41±6.26	0.412
Visceral Adiposity Index	1.57±0.75	2.76±1.47	<0.001
HbA1c (%)	6.83±0.59	6.97±0.66	0.285
HbA1c (mmol/mol)	51±6.4	53±7.2	0.285
HOMA-IR	2.46±2.59	3.53±3.51	0.099
Fasting Insulin (pmol/l)	54.40±60.47	72.06±71.14	0.208
Fasting C-peptide (nmol/l)	0.13±0.10	0.16±0.18	0.229
Fasting glucose (mmol/l)	7.36±1.27	7.69±1.69	0.292
2 h after breakfast glucose (mmol/l)	6.79±1.07	6.86±1.74	0.806
2 h after lunch glucose (mmol/l)	7.51±1.41	7.59±1.96	0.821
2 h after dinner glucose (mmol/l)	7.31±1.74	7.98±1.98	0.093
Urinary Albumin-to-Creatinine Ratio (UACR) (mg/g)	19.83±55.35	14.41±23.15	0.581

Student’s t Test after testing for equality of variance (Levene test).

### ROC Curve Analysis

ROC curve analysis was performed for BMI, WC, HC, WHR, BAI and VAI to evaluate their diagnostic performance for predicting an HATD (Table 4).

**Table 4:** Optimal cut-off points of the various anthropometric indices (BMI, WC, HC, WHR, BAI, VAI) to detect type 2 diabetic patients with an “hypothetical adipose tissue dysfunction” (Cluster 2).

	Cutoff Point	Sens.	Spec.	Area under ROC curve	SE	95% CI	p
		(%)	(%)				
**BMI**	32	30.91	86.11	0.588	0.060	0.48–0.69	0.142
**WC**	106	41.82	77.78	0.573	0.060	0.46–0.67	0.230
**HC**	100	30.91	80.56	0.507	0.062	0.40–0.61	0.916
**WHR**	0.96	52.73	66.67	0.574	0.060	0.46–0.67	0.225
**BAI**	37.38	32.73	83.33	0.537	0.061	0.42–0.64	0.552
**VAI**	2.00	65.45	80.56	0.767	0.048	0.66–0.84	<0.001

BMI: Body Mass Index; WC: Waist Circumference; HC: Hip Circumference; WHR: Waist/Hip ratio; BAI: Body Adiposity Index; VAI: Visceral adiposity Index. Sens. (sensitivity); Spec. (Specificity); SE (Standard Error).

**Figure 2:**
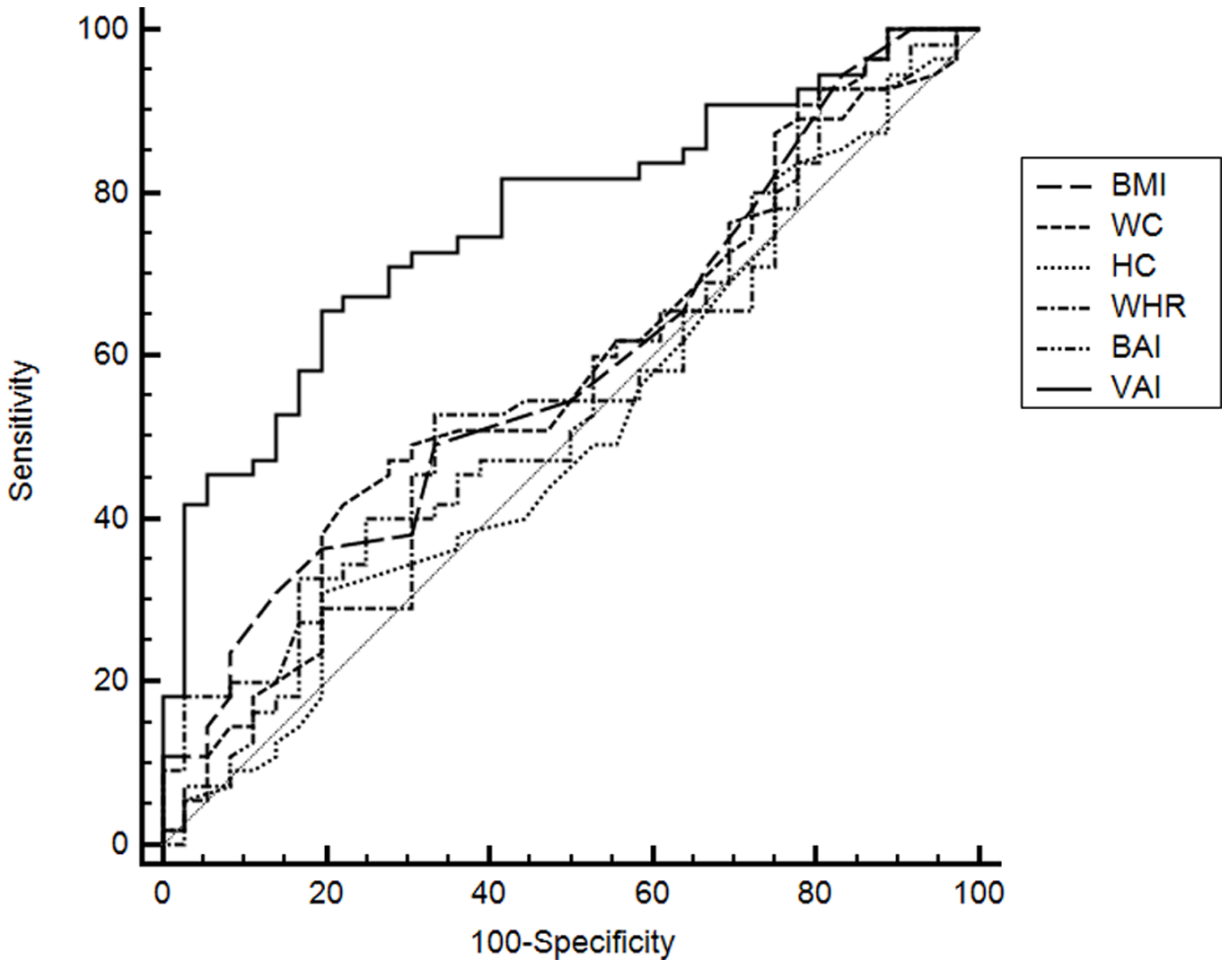
ROC Curves of the various anthropometric indices (BMI, WC, HC, WHR, BAI, VAI) for “hypothetical adipose tissue dysfunction” (Cluster 2).

C-statistics were then compared to identify which parameter was a better indicator for HATD. The C-statistics were 0.588 (95% CI 0.48–0.69) for BMI, 0.573 (95% CI 0.46–0.67) for WC, 0.507 (95% CI 0.40–0.61) for HC, and 0.574 (95% CI 0.46–0.67) for WHR, 0.537 (95% CI 0.42–0.64) for BAI and 0.767 (95% CI 0.66–0.84) for VAI. VAI showed a significantly higher C-statistic compared to BMI (difference: 0.178; 95% CI: 0.03–0.32; SE: 0.07; p = 0.014), WC (difference: 0.194; 95% CI: 0.04–0.34; SE: 0.07; p = 0.009), HC (difference: 0.260; 95% CI: 0.11–0.40; SE: 0.07; p<0.001), WHR (difference: 0.193; 95% CI: 0.04–0.33; SE: 0.07; p = 0.009) and BAI (difference: 0.230; 95% CI: 0.08–0.37; SE: 0.07; p = 0.001) (figure 2). No difference in C-statistic was found among the other anthropometric indices.

## Conclusions

We demonstrate in this study that the VAI is a good indicator of an altered adipokine profile and its associated cardiometabolic risk.

The emerging paradigm of ATD supports the view that adipose tissue dysregulation (altered fat distribution and function) might play a crucial role in the pathogenesis of insulin resistance and atherosclerosis. From this viewpoint, atherosclerotic cardiovascular disease and DM2 seem to arise from a “common soil”, characterized by altered adipocytokine production, increase in the lipolytic activity of adipose tissue and low-grade inflammation [24]–[26]. In addition, recently the hypothesis that subcutaneous AT has a limited capacity to increase its mass safely has been gaining increasing recognition [27], [28]. This capacity is individually determined by a wide range of environmental and genetic factors. The absence of adaptive hyperplasia of the subcutaneous adipose tissue could be associated with visceral obesity and excess ectopic fat.

We studied the ability of various common anthropometric indices to correlate with a wide panel of adipocytokines. Though the literature data show a continued involvement of new adipocytokines, we chose to study some of those that have more scientific evidence about their ability to express a condition of ATD [29], [30].

In our DM2 patients there were no gender differences except as regards the leptinemic system: leptin levels are higher in women than in men and the level of its soluble receptor is inversely proportional; this known datum could be an expression of a state of relative leptin resistance in women with diabetes [31]. Also supporting this hypothesis is the observation of higher levels of NEFA in women, which may reflect the reduction of anti-lipotoxic activity carried out by leptin [32].

Among all the anthropometric indexes studied the VAI is the one that showed the greatest number of correlations with the adipocytokines and the lipids (including NEFA). The VAI was able to express both the altered endocrine function of adipose tissue and the state of relative leptin resistance and low-grade inflammation, which are all alterations present in a state of ATD. Particularly strong is the correlation with visfatin (adipocytokine prevalently secreted by visceral adipose tissue, which according to some authors may have adipogenic effects and is a good candidate to explain the accumulation of visceral adipose tissue associated with insulin resistance) [33], [34], resistin (a potential mediator of obesity related insulin resistance in rodents, but still today a subject of controversy in human obesity and human epidemiologic studies) [35] and hs-CRP (systemic markers of inflammation, which have been found to independently predict future coronary heart disease in type 2 diabetes) [36]. Furthermore, among all the indices it was the only one that showed an inverse correlation with adiponectin. This datum, also confirmed in other studies [9], [17], [21], is of major significance, given that the adiponectin reduction is a key player in the development of cardiovascular complication in DM2, because it is the only known protective adipocytokine with insulin-sensitizing, anti-inflammatory and anti-atherogenic properties [29], [37], [38]. Lastly, it is also worth noting the significant association of VAI with VEGF and HGF, an expression of the fact that activated adipocytes produce multiple angiogenic factors including leptin, HGF, VEGF, which stimulate neovascularization during fat mass expansion [39].

However, the primary difficulty of our study was to test the diagnostic ability of the various anthropometric indices towards a clinical entity such as ATD, which to date has still not been univocally framed. For these reasons we used the two-step cluster analysis, to identify the patients with the worst adipocytokines profile, and use cautiously the diction “hypothetical adipose tissue dysfunction”. A limitation of our study, however, consists in the fact that since all patients have a DM2, though not in a phase of glycemic decompensation, it is assumed that all have a certain degree of ATD. This datum is also apparent from the fact that, despite the differences in the adipocitokine levels between the two clusters, there were no significant differences regarding the glycemic parameters (HbA1c, HOMA IR, glycemic profile), as if the ATD only concerned the nonglycemic aspects of insulin-resistance. Probably, the overlap between the two groups for glycemic control and insulin sensitivity, despite the different adipocytokine profile, it could also be due to extrahepatic actions of metformin, able to activate AMP-activated protein kinase and reduce acetyl-CoA carboxylase protein levels in human adipose tissue in vivo [40]. The utility of the VAI in patients with diabetes does not lie in the ability to assess the cardiovascular risk linked to glucose toxicity (for these aspects the most important parameter is always HbA1c), but it might mirror the residual cardiovascular risk despite the normalization of glycemic, lipid and hemodynamic parameters [41]. Finally, in our study we also identified a precise cut-off of the VAI able to identify a HATD. This cut-off of 2 seems to be comparable to the cut-off point identified, in the same age range, in a previous study (in which in the general population an ATD dysfunction is hypothesized in subjects with metabolic syndrome) [10].

In conclusion, although still lacking prospective studies that can attribute to the VAI a prognostic role on cardiovascular risk, given the simplicity of WC and BMI measurement and Triglycerides and HDL Cholesterol assessment, we suggest that the VAI would be an easy tool for the evaluation of the cardiometabolic risk in type 2 diabetes or in other populations, mainly in the absence of an overt metabolic syndrome.
